# The effect of cost-sharing in private health insurance on the utilization of health care services between private insurance purchasers and non-purchasers: a study of the Korean health panel survey (2008–2012)

**DOI:** 10.1186/s12913-015-1153-0

**Published:** 2015-10-28

**Authors:** Young Choi, Jae-Hyun Kim, Ki-Bong Yoo, Kyoung Hee Cho, Jae-Woo Choi, Tae Hoon Lee, Woorim Kim, Eun-Cheol Park

**Affiliations:** 1 Department of Public Health, Graduate School, Yonsei University, Seoul, Korea; 2 College of Medicine, Institute of Health Services Research, Yonsei University, Seoul, Korea; 3 Department of Preventive Medicine and Institute of Health Services Research, College of Medicine, Yonsei University, 50 Yonsei-ro, Seodaemun-gu, Seoul, 120-752 Korea; 4 Department of Healthcare Management, Eulji University, Seongnam, Korea

**Keywords:** Cost-sharing, Private health insurance, Indemnity health insurance, Health care utilization

## Abstract

**Background:**

Private health insurance in South Korea mainly functions as supplementary and complementary health insurance that compensates for insufficient coverage by National Health Insurance. However, full private coverage of public sector cost-sharing led to the problem of encouraging moral hazard–induced utilization, resulting in a policy change that occurred in October 2009. At that time, the Korean government introduced a minimum cost-sharing policy for indemnity health insurance. The purpose of this study was to analyze the effect of cost-sharing in private health insurance on health care utilization.

**Methods:**

We analyzed data collected from the Korean Health Panel Survey from October 2008 to December 2011. We restricted the two groups to 803 purchasers with indemnity health insurance and 7023 non-purchasers who did not obtain any private health insurance. A difference-in-difference analysis was used to evaluate the effect of the 2009 policy.

**Results:**

After the policy change, the utilization of outpatient visits by purchasers gradually decreased more than non-purchasers (0.015 in 2009 [*p =* 0.758], −0.117 in 2010 [*p* < 0.016], and −0.140 in 2011 [*p* = 0.004]). However, utilization of inpatient services was not statistically significant. Notably, the magnitude of the cost-sharing effect in indemnity health insurance was stronger for those receiving medical aid. Among this group, utilization of outpatient services (after the policy change in 2009) decreased more so than non-purchasers. Patients with three or more chronic diseases have not changed their health care utilization.

**Conclusions:**

Our results implied meaningful lessons for decision-makers and future health insurance policies in Korea and other countries in terms of cost-sharing in medical care. When policy makers intend to implement the cost-sharing, a different copayment scheme is needed according to the socioeconomic status or disease severity.

## Background

The primary framework for the South Korean health insurance system is the National Health Insurance (NHI) program. The NHI program was implemented in 1963 by the Medical Insurance Act to provide a social safety net for the health care needs of all Korean citizens. In 1977, mandatory participation in the NHI program was applied to firms with 500 or more employees and has gradually been extended to public officials. By 1989, virtually all citizens in South Korean territories were subject to compulsory enrollment in the NHI program [1].

Although Korea accomplished universal coverage through this mandatory NHI program, the public sector accounts for only 55.3 % of total health expenditure. As of 2011, this figure was well below the average of 72.2 % for Organization for Economic Co-operation and Development (OECD) countries [2]. Moreover, household expenditures for health constitutes 35.2 % of total household expenditures, which is greater than the average of 19.6 % for OECD countries [2]. As a consequence of such low coverage and high household spending, Korean people have had a tendency to solve insufficient coverage needs by using private health insurance (PHI) [3, 4].

Colombo and Tapay [5] emphasized that PHI is distinguished as primary, duplicate, complementary, and supplementary health insurance based on its role within the health care system and its interaction with public coverage. In South Korea, PHI mainly functions as supplementary and complementary health insurance under the NHI program [6]. There are two types of PHI in Korea. One type is fixed benefit insurance, which pays a fixed amount defined in accordance with the PHI contract. The other type is indemnity health insurance, which fully covers services uninsured by the NHI program and out-of-pocket payments for services covered by the NHI program [1, 4]. However, until October 2009, Korea faced the problem of patients with indemnity health insurance either paying very little or not participating in cost-sharing whatsoever. An OECD report claimed that complementary health insurance helps ensure access to needed care. However, full private coverage of public sector cost-sharing encourages moral hazard–induced utilization [5]. Unless some form of cost-sharing is retained to maintain individual cost awareness, PHI coverage hinders efforts to control outlays from public systems [5].

According to the Korea Health Panel Survey, the total percentage of households with PHI increased from 70.6 % in 2008–76.8 % in 2011 [7]. With this increasing number of PHI purchasers, the Korean government has been concerned about the potential negative effects of PHI on NHI. If a purchaser of PHI utilizes more health care (due to the decrease in copayment under NHI), then PHI has a fiscal spillover on NHI (i.e., PHI may result to higher health care utilization and spending), and there is an inequity in health care utilization between the purchasers and non-purchasers of PHI. Several studies have shown concordant opinion that medical utilization of those who purchase PHI is higher than those without PHI [6, 8, 9]. In addition, a study showed that those with indemnity health insurance utilize more health care than those who purchase PHI with fixed benefits [10]. Consequently, complementary PHI is likely to contribute to the rapid increase in health expenditure, inducing fragmentation of the health system [4].

For these reasons, the Korean government introduced minimum cost-sharing for indemnity health insurance in October 2009. Before the policy change, private health insurers almost fully covered the total amount of out-of-pocket payments after NHI for inpatient care to purchasers with indemnity insurance. The deductible per outpatient visit varied from approximately 5000–10,000 Korean Won (exchange rate: 1 USD = 1102 KRW; 1 EUR = 750 KRW, USD $ 5–9; EUR € 6–13). This new policy requires that if inpatient expenditures are 2,000,000 KRW (USD $ 1,813; EUR € 2,666) or less per year, purchasers with indemnity health insurance should pay 10 % of the out-of-pocket payments after NHI (i.e., coverage by indemnity health insurance was reduced from 100–90 %). If expenditures were 2,000,000 KRW (USD $ 1,813; EUR € 2,666) or more per year, purchasers could still be paid 100 % of the inpatient expenditures after NHI from their PHI company. For outpatient care, deductible costs were changed by 10,000 KRW (USD $ 9; EUR € 13) per clinic visit, 15,000 KRW (USD $ 14; EUR € 20) per hospital visit, and 20,000 KRW (USD $ 18; EUR € 27) per visit to a general hospital.

The introduction of cost-sharing in indemnity health insurance was designed to restrict health care expenditures by reducing moral hazard in medical utilization. However, there are two side effects of copayments. Sharing the cost of health care services should discourage moral hazard in health care consumers, and the over-utilization of medical services can be expected to decline [11–13]. However, patients with low income status may receive insufficient care when required due to cost-sharing [14, 15]. The role of cost-sharing in health care utilization has been extensively examined. Studies performed in other countries found that out-of-pocket payments on health care utilization had the effect of controlling health care utilization for outpatient and inpatient care, as well as for prescription drugs [16–26]. In Korea, the number of physician visits decreased as a result of increasing copayments [27]. A study found that patients with low income and users of clinics were more sensitive to cost-sharing for ambulatory utilization [28]. Regarding cost-sharing among the elderly, a study found that outpatient cost-sharing for this population had little effect on controlling health care utilization [29]. Additionally, a recent study showed that the effect of the copayment waiver policy on children under the age of 6 years was unclear [30]. However, the effect of cost-sharing in indemnity health insurance under NHI has not been studied.

The purpose of this study was to analyze the effect of cost-sharing in PHI (indemnity) on health care utilization by examining the number of outpatient visits, the number of inpatient visits, and length of stay. The cost-sharing included only the beneficiaries of indemnity health insurance. Thus, this study compared the difference between those with indemnity health insurance and those without any PHI and aimed to reveal the net effect of the cost-sharing policy on individuals with indemnity health insurance. Specifically, this study tested the following two hypotheses: (1) After the introduction of cost-sharing, those with indemnity health insurance are less likely to use health care services than those without PHI over time. If so, (2) the magnitude of the cost-sharing effect differs according to public health insurance status (i.e., Medical Aid or National Health Insurance).

## Methods

### Data and study population

Data was acquired from the Korean Health Panel Survey, which was collected from October 2008 to December 2011. Detailed information for families and individuals was collected from a nationally representative sample of households; variables included demographic characteristics, income, savings and expenses, employment, housing, chronic conditions, use of medical services, medications, charges and source of payments, PHI, pregnancy and delivery, elder care, health behaviors, and health awareness [31].

The initial 2008 baseline data consisted of information from 21,283 individuals in 7009 households. The control group included those who purchased any PHI, and the intervention group (i.e., beneficiary group) comprised neither those who purchased PHI with fixed benefits nor those who purchased both fixed-benefit and indemnity PHI, to reduce the heterogeneity of the intervention group. Thus, we restricted the study population to individuals who purchased indemnity health insurance or did not purchase any PHI. Among the 21,283 individuals at baseline (2008), we eliminated 12,168 participants who purchased PHI with fixed benefits, as well as 1290 who purchased PHI with both fixed benefits and indemnity. Among 7825 individuals, we excluded one participant whose employment status was unavailable at baseline. Overall, the sample used for the analysis was restricted to 7021 non-purchasers and 803 purchasers.

We respected the provisions of the Declaration of Helsinki and obeyed the protocol for the research project, adhering to safe and ethical principles. As this study used open national data for public access, we did not need to obtain individual informed consent.

### Statistical analysis

We compared the general characteristics between purchasers and non-purchasers at baseline (2008) and used independent t-tests to compare the means of the health care utilization variables. The primary statistical model was difference-in-difference analysis (DID). The DID was used to reduce the probabilities of time-invariant omitted variables and time trends by comparing results before and after policy reform [32]. DID analysis is one of the most frequently used and informative study designs for examining the effects of interventions in the social sciences. To briefly introduce DID analysis, consider an intervention given at some time point between time *a* and time *b*. There are only two groups (*r* = 0 or 1), and the intervention is given only to group 1. For an individual *i* with responses y_*ia*_ and y_*ib*_, *E* (y_*ib*_ – y_*ia*_ | *r* = 0) includes only the time effect, whereas *E* (y_*ib*_ – y_*ia*_ | *r* = 1) includes both the time and intervention effects. Thus:

$$ \mathrm{DID}=\mathrm{E}\left({\mathrm{y}}_{ib}-{\mathrm{y}}_{ia}\left|\right.r=1\right)-\mathrm{E}\left({\mathrm{y}}_{ib}-{\mathrm{y}}_{ia}\left|\right.r=0\right) $$

identifies the desired intervention effect (or the net effect of policy reform) by removing the time effect, where the subscript *i* is omitted assuming independent and identical distribution across *i* = 1, *N* [33].

A regression equation was used to estimate the intervention effect of the policy. The equation is shown below:

$$ {\mathrm{Y}}_{\mathrm{i}}=\mathrm{a}+{\upbeta}_1\mathrm{G}+{\upbeta}_2\mathrm{T}+{\upbeta}_3\mathrm{G}\times \mathrm{T}+{\upbeta}_4\mathrm{X}+\upvarepsilon $$

In this equation, Y_i_ is the total number of outpatient visits per person, the total number of admission days per person, or length of stay per person. Indices of medical use were analyzed using a negative binomial model with a log link function because the data in this study were not normally distributed. Thus, we used the negative binomial model by using Akaike Information Criterion. To select the appropriate model, we compared the regression with poisson distribution and negative binomial model. G is the group variable (non-purchaser or purchaser), T is the time variable (Y2008: pre-policy change; Y2009: time of policy change; Y2010: 1 year after policy change; Y2011: 2 years after policy change), and X is the set of covariates (i.e., demographic, socioeconomic, and health-related factors). In the regression, β_3_ was the estimator of the difference-in-difference estimator (the net intervention effect of cost-sharing). Additionally, we analyzed the effects of cost sharing on helath care utilization according to public health insurance status (i.e., Medical Aid or National Health Insurance) and the number of disease. All statistical analyses were performed using SAS version 9.2 (SAS Institute, Inc., Cary, NC, USA). The Korean Won was converted to US dollars according to the 2008 average exchange rate: 1 USD = 1101 KRW; 1 EUR = 750 KRW [34].

### Variables

The main independent variable was the interaction term (difference-in-difference estimator) between the group (purchaser or non-purchaser) and time (2008–2011). The dependent variables measured quantity of health care utilization (i.e., the total number of outpatient visits per person, the total number of admission days per person, and length of stay per person).

Demographic, socioeconomic, and health-related factors were included in this study. Demographic factors included sex, age (1–19, 20–39, 40–59, above 60), marital status (married, divorced/widowed/unmarried), and region (rural, urban). Indicators of socioeconomic status included educational level (elementary school or below, middle school, high school, or college or above), public health financing (National Health Insurance, Medical Aid), household income (adjusted by the square root of household numbers), and employment status (employed, unemployed), Health-related factors included the number of chronic diseases (0, 1, 2, or ≥3 diseases).

## Results

Table 1 presents the results for the general characteristics between non-purchasers and purchasers at the baseline (2008). There were 7824 total participants (7021 non-purchasers and 803 purchasers).

**Table 1:** General characteristics of both non-purchasers and purchasers at baseline (2008)

					Unit: N, %
Variable	Total	Non-purchasers		Purchasers	
Total	7824	7021	(89.7)	803	(10.3)
Sex					
Man	3839	3434	(48.9)	405	(50.4)
Woman	3985	3587	(51.1)	398	(49.6)
Age					
1–19	1752	1317	(18.8)	435	(54.2)
20–39	1699	1525	(21.7)	174	(21.7)
40–59	1539	1421	(20.2)	118	(14.7)
≥60	2834	2758	(39.3)	76	(9.5)
Marital status					
Married	4107	3556	(50.6)	551	(68.6)
Divorced/widowed/unmarried	3717	3465	(49.4)	252	(31.4)
Region					
Rural	4333	3875	(55.2)	458	(57.0)
Urban	3491	3416	(44.8)	345	(43.0)
Educational level					
Elementary school or below	3080	2711	(38.6)	369	(46.0)
Middle school	1071	972	(13.8)	99	(12.3)
High school	1917	1743	(24.8)	174	(21.7)
College or above	1756	1595	(22.7)	161	(20.0)
Public health financing					
NHI (National Health Insurance)	7225	6444	(91.8)	781	(97.3)
Medical Aid (for the poor)	599	577	(8.2)	22	(2.7)
Household Income					
Q1 (High)	1392	1169	(16.7)	223	(27.8)
Q2	1686	1418	(20.2)	268	(33.4)
Q3	2256	2052	(29.2)	204	(25.4)
Q4 (Low)	2490	2382	(33.9)	108	(13.4)
Employment status					
Employed or self-employed	4338	3755	(53.5)	583	(72.6)
Unemployed or inactive	3486	3266	(46.5)	220	(27.4)
Chronic disease					
None	4109	3537	(50.4)	572	(71.2)
One	1503	1358	(19.3)	145	(18.1)
Two	936	899	(12.8)	37	(4.6)
Three or more	1276	1227	(17.5)	49	(6.1)

Table 2 presents the results of change in health care utilization (i.e., outpatient visits per person, inpatient admission per person, and length of stay per person) between non-purchasers and purchasers from 2008–2011. While the mean number of outpatient visits in the non-purchaser group gradually increased (13.80 in 2008, 16.10 in 2009, 18.70 in 2010, and 20.86 in 2011), the mean number of outpatient visits in the purchaser group slowly increased (11.78 in 2008, 11.86 in 2009, 12.24 in 2010, and 12.32 in 2011). The trend of inpatient utilization for both purchaser and non-purchaser increased until 2010. The average admission of those with indemnity health insurance decreased in 2011, whereas utilization by those without any PHI remained increased. The average length of stay per person gradually increased from 2008–2010 and then decreased in 2011 for both those with indemnity PHI and those without any PHI.

**Table 2:** Changes in health care utilization between non-purchasers and purchasers from 2008–2011

Year	Outpatient visits per person			Inpatient admissions per person			Length of stay per person		
	Purchasers (*n* = 803)	Non-purchasers (*n* = 7021)	*p*-value	Purchasers (*n* = 803)	Non-purchasers (*n* = 7021)	*p*-value	Purchasers (*n* = 803)	Non-purchasers (*n* = 7021)	*p*-value
2008	11.78	13.80	0.001	0.12	0.16	0.009	0.92	2.26	<.0001
2009	11.86	16.10	<.0001	0.13	0.17	<.0001	0.96	2.66	<.0001
2010	12.24	18.70	<.0001	0.14	0.21	<.0001	1.21	3.42	<.0001
2011	12.32	20.86	<.0001	0.12	0.23	<.0001	0.96	3.21	<.0001

Table 3 shows the results of the effect of cost-sharing in indemnity health insurance on health care utilization. With respect to the utilization of outpatient visits, those who purchased indemnity PHI utilized more than those who did not purchase any PHI (2.599; *p* < 0.000). For all participants, the utilization of outpatient visits increased over time. The result from the interaction coefficient (difference-in-difference estimator) indicated the net effect of policy change. After the policy change in 2009, purchaser utilization of outpatient services per person (difference in difference estimator) was −0.117 in 2010 (*p* = 0.0045) and −0.140 in 2011 (*p* = 0.004). These values indicate that the degree of decrease among those with indemnity PHI after policy implementation was greater than that of those without any PHI. The number of inpatient visits and length of stay per person showed a similar trend; the results for the number of inpatient visits and length of stay per person were not statistically significant.

**Table 3:** Effects of cost-sharing in indemnity health insurance on health care utilization

Variable	Outpatient visits per person			Inpatient admissions per person			Length of stay per person		
	ß	S.E	*p-value*	ß	S.E	*p-value*	ß	S.E	*p-value*
Sex									
Man	Ref.			Ref.			Ref.		
Woman	0.179	0.025	<.0001	−0.206	0.060	0.001	−0.356	0.093	0.000
Age									
1–19	Ref.			Ref.			Ref.		
20–39	−0.592	0.041	<.0001	0.272	0.145	0.060	0.675	0.200	0.001
40–59	−0.426	0.049	<.0001	0.224	0.166	0.177	1.381	0.243	<.0001
above 60	−0.114	0.042	0.007	0.486	0.168	0.004	1.592	0.191	<.0001
Marital status									
Divorced/widowed/unmarried	Ref.			Ref.			Ref.		
Married	0.371	0.032	<.0001	0.261	0.066	<.0001	−0.068	0.147	0.646
Educational level									
Below elementary school	Ref.			Ref.			Ref.		
Middle school	−0.437	0.030	<.0001	−0.244	0.088	0.006	−0.317	0.126	0.012
High school	−0.573	0.031	<.0001	−0.284	0.074	0.000	−0.388	0.122	0.001
Above college	−0.790	0.040	<.0001	−0.515	0.109	<.0001	−0.670	0.204	0.001
Public health financing									
National Health Insurance	Ref.			Ref.			Ref.		
Medical Aid (for the poor)	0.245	0.037	<.0001	0.593	0.083	<.0001	0.770	0.139	<.0001
Chronic disease									
None	Ref.			Ref.			Ref.		
One	0.818	0.032	<.0001	0.801	0.114	<.0001	1.056	0.148	<.0001
Two	1.133	0.036	<.0001	0.970	0.103	<.0001	1.089	0.186	<.0001
Three or more	1.478	0.035	<.0001	1.418	0.097	<.0001	1.340	0.150	<.0001
Household Income									
Q1(High)	Ref.			Ref.			Ref.		
Q2	−0.061	0.027	0.023	0.093	0.093	0.316	−0.011	0.115	0.926
Q3	−0.053	0.027	0.048	−0.037	0.080	0.639	−0.079	0.122	0.516
Q4(Low)	−0.088	0.028	0.002	−0.150	0.080	0.062	−0.137	0.139	0.324
Employment status									
Employed or self-employed	Ref.			Ref.			Ref.		
Unemployed or inactive	0.116	0.025	<.0001	0.320	0.055	<.0001	0.445	0.099	<.0001
Region									
Rural	Ref.			Ref.			Ref.		
Urban	−0.016	0.023	0.475	−0.296	0.055	<.0001	−0.293	0.087	0.001
Time									
2008	Ref.			Ref.			Ref.		
2009	0.050	0.016	0.002	−0.073	0.067	0.281	0.124	0.113	0.269
2010	0.115	0.019	<.0001	0.020	0.067	0.769	0.036	0.096	0.708
2011	0.142	0.020	<.0001	0.047	0.071	0.505	−0.038	0.097	0.695
Group (indemnity health insurance)									
Non-purchasers	Ref.			Ref.			Ref.		
Purchasers	0.340	0.042	<.0001	0.320	0.153	0.036	0.202	0.184	0.272
Time x Group (difference in difference estimator)									
2009	0.015	0.050	0.758	0.154	0.173	0.372	0.108	0.242	0.655
2010	−0.117	0.049	0.016	0.045	0.167	0.785	0.264	0.257	0.304
2011	−0.140	0.049	0.004	−0.209	0.169	0.216	−0.147	0.224	0.511

Additionally, results of the cost-sharing effect on health care utilization by type of health insurance (i.e., Medical Aid for the poor and national health insurance) are shown in Table 4. Among those receiving Medical Aid, purchaser utilization of outpatient services per person (difference in difference estimator) was −0.491 in 2010 (*p* = 0.025) and −0.580 in 2011 (*p* = 0.008) after the policy change in 2009. Among the national health insurance beneficiaries, utilization of outpatient services was −0.098 in 2010 (*p* = 0.053) and −0.115 in 2011 (*p* = 0.025). These results indicate that the magnitude of the effect of cost-sharing in indemnity health insurance was strong for Medical Aid.

**Table 4:** Effects of cost-sharing in indemnity health insurance on health care utilization according to public health financing

	Medical aid (for the poor, *n* = 599)									National helath insurance (*n* = 7225)								
Variable^a^	Outpatient visits per person			Inpatient admissions per person			Length of stay per person			Outpatient visits per person			Inpatient admissions per person			Length of stay per person		
	ß	S.E	*p-value*	ß	S.E	*p-value*	ß	S.E	*p-value*	ß	S.E	*p-value*	ß	S.E	*p-value*	ß	S.E	*p-value*
Time																		
2008	Ref.			Ref.			Ref.			Ref.			Ref.			Ref.		
2009	0.071	0.044	0.105	−0.016	0.135	0.905	−0.025	0.273	0.927	0.047	0.017	0.006	−0.083	0.075	0.269	0.157	1.280	0.201
2010	0.086	0.050	0.084	0.040	0.134	0.762	0.027	0.197	0.889	0.121	0.021	<.0001	0.010	0.075	0.894	0.020	0.190	0.851
2011	0.092	0.050	0.069	0.057	0.140	0.682	−0.092	0.228	0.688	0.146	0.022	<.0001	0.043	0.080	0.588	−0.017	−0.160	0.873
Group (indemnity health insurance)																		
Non-purchasers	Ref.			Ref.			Ref.			Ref.			Ref.			Ref.		
Purchasers	0.928	0.207	<.0001	1.557	0.518	0.003	1.898	0.649	0.003	0.312	0.043	<.0001	0.250	0.161	0.119	0.128	0.680	0.498
Time x Group (difference-in-difference estimator)																		
2009	−0.345	0.180	0.055	−0.690	0.643	0.283	−1.613	0.718	0.025	0.038	0.053	0.469	0.180	0.178	0.312	0.147	0.590	0.556
2010	−0.491	0.219	0.025	−1.695	0.738	0.022	−2.978	0.910	0.001	−0.098	0.051	0.053	0.153	0.172	0.373	0.393	1.520	0.129
2011	−0.580	0.218	0.008	−0.966	0.542	0.074	−1.435	0.682	0.035	−0.115	0.051	0.025	−0.181	0.179	0.313	−0.104	−0.450	0.653

^a^Adjusted for sex, age, marital status, education level, the number of chronic disease, household income, region, and employment status

The results of the cost-sharing effect on health care utilization by the number of disease are shown in Table 5. Among those with no chronic disease or one to two diseases, purchaser utilization of outpatient services per person decreased in 2010 and 2011. However, purchaser utilization of outpatient services per person has not changed their health care utilization.

**Table 5:** Effects of cost-sharing in indemity health insurance on health care utilization according to the number of disease

Variable^a^	Outpatient visits per person			Inpatient admissions per person			Length of stay per person		
	ß	S.E	*p-value*	ß	S.E	*p-value*	ß	S.E	*p-value*
No chronic disease (*n* = 4109)									
Time									
2008	Ref.			Ref.			Ref.		
2009	0.227	0.038	<.0001	0.136	0.131	0.297	0.344	0.217	0.112
2010	0.366	0.042	<.0001	0.351	0.144	0.015	0.081	0.192	0.672
2011	0.427	0.046	<.0001	0.229	0.147	0.118	0.136	0.202	0.500
Group (indemnity health insurance)									
Non-purchaser	Ref.			Ref.			Ref.		
Purchaser	0.444	0.062	<.0001	0.697	0.188	0.000	0.455	0.234	0.052
Time x Group (difference in difference estimator)									
2009	0.029	0.079	0.716	−0.126	0.251	0.617	0.037	0.343	0.915
2010	−0.160	0.074	0.031	−0.180	0.257	0.483	0.470	0.344	0.171
2011	−0.177	0.079	0.025	−0.347	0.263	0.187	−0.144	0.328	0.660
1–2 (*n* = 2439)									
Time									
2008	Ref.			Ref.			Ref.		
2009	0.015	0.023	0.519	−0.155	0.126	0.216	0.074	0.198	0.709
2010	0.054	0.030	0.075	−0.079	0.127	0.537	−0.095	0.146	0.515
2011	0.077	0.034	0.022	−0.034	0.158	0.830	−0.148	0.159	0.353
Group (indemnity health insurance)									
Non-purchaser	Ref.			Ref.			Ref.		
Purchaser	0.267	0.066	<.0001	0.095	0.291	0.744	0.190	0.376	0.613
Time x Group (difference in difference estimator)									
2009	−0.226	0.069	0.001	0.403	0.295	0.172	−0.059	0.443	0.894
2010	−0.245	0.075	0.001	0.122	0.271	0.654	−0.104	0.415	0.802
2011	−0.313	0.079	<.0001	−0.134	0.291	0.645	−0.258	0.420	0.539
3 or more (*n* = 1276)									
Time									
2008	Ref.			Ref.			Ref.		
2009	0.019	0.022	0.385	−0.083	0.088	0.345	−0.168	0.136	0.217
2010	0.075	0.024	0.002	−0.020	0.082	0.808	−0.013	0.138	0.926
2011	0.116	0.025	<.0001	0.085	0.083	0.306	0.022	0.136	0.871
Group (indemnity health insurance)									
Non-purchaser	Ref.			Ref.			Ref.		
Purchaser	−0.093	0.126	0.459	−0.385	0.366	0.293	−0.073	0.414	0.861
Time x Group (difference in difference estimator)									
2009	−0.014	0.122	0.909	−0.021	0.428	0.962	−0.795	0.515	0.123
2010	0.000	0.148	0.998	0.077	0.429	0.858	−0.266	0.444	0.549
2011	−0.020	0.131	0.878	0.364	0.434	0.402	0.445	0.500	0.374

^a^Adjusted for sex, age, marital status, education level, the number of chronic disease, household income, region, and employment status

## Discussion

The aim of this study was to examine the effect of cost-sharing in PHI (indemnity) on the utilization of health care. We compared a group of individuals that purchased indemnity health insurance with a group of individuals that did not purchase any PHI. We analyzed the differences in utilization of inpatient and outpatient visits and length of stay per person between the two groups (before and after policy reform). After the introduction of cost-sharing, purchaser utilization of outpatient visits decreased more than non-purchaser utilization of outpatient visits over time. However, the magnitude of the cost-sharing effect was stronger for the poor (i.e., those receiving Medical Aid).

Our results were similar to the results of several previous studies. In France, reduction of out-of-pocket payments was found to encourage moral hazard in home-visit care [35]. A study of self-selection and moral hazard in Chile found that cost-sharing significantly affected outpatient utilization yet not inpatient utilization [36]. However, the findings of a study performed in Korea were different from our findings. This study used data from a PHI company and analyzed changes in health care consumption before and after the introduction and expansion of cost-sharing in indemnity PHI. The post-introduction and expansion group consumed less health care than the pre-introduction and expansion group for both inpatient days and outpatient visits [37]. With regard to the inpatients health care, our findings may be different from those of this previous study. In our study, cost-sharing for inpatient health care did not reduce the utilization of inpatient visits or length of stay per person. Differences in the mechanisms between outpatient and inpatient visits may have contributed to the differences in the results. Inpatient-care utilization is influenced more by physician recommendations and disease severity than by patient decisions [17]. In other words, the role of the medical provider as decision-maker was more important than the role of the patient in the case of inpatient utilization. Therefore, cost-sharing for inpatient health care utilization was not affected. Another possible explanation is because the data used in the previous study in Korea was obtained from only one PHI company.

The introduction of cost-sharing in indemnity health insurance appeared to have affected the decrease in outpatient visits. In 2009, the outpatient visits of purchasers declined more than non-purchasers; however, this difference was not statistically significant. It is possible that the effect size was not significant due to the policy only being implemented in October 2009. Nevertheless, the number of outpatient visits gradually declined from 2009–2011. This result indicated that the effect of cost-sharing on the utilization of inpatient care occurred after policy reform. The results of previous studies found that individuals who purchased PHI had an influence on increases in health care utilization [4, 6, 10, 38]. Consequently, our results may indicate that PHI-purchaser outpatient visits occurred after the policy change.

Additionally, we analyzed the effect of cost-sharing on health care utilization by type of health insurance (Medical Aid, NHI). The utilization of outpatient visits declined for both Medical Aid and NHI beneficiaries after the introduction of cost-sharing. However, the magnitude of the cost-sharing effect was stronger for Medical Aid. This result may indicate that low-income patients are more sensitive to cost-sharing. Other studies reported similar differences in the effect size of cost-sharing by economic status. A study in Japan showed that utilization of outpatient care was most sensitive to the copayment rate [16]. Per-capita income stratification models revealed that the greatest copayment effect on inpatient care was for the lowest income group in the National Health Insurance system. A Korean study also showed that the magnitude of the impact of copayment on the number of physician visits varied depending on income level. Increasing cost-sharing rates affected health care utilization by individuals with relatively low income [28]. These results indicated that increasing copayment rates may raise problems by discouraging access to medical care services for the poor.

We additionally analyzed the effect of cost-sharing on health care utilization by the number of diseases to determine how different patterns of utilization. In our study, the patterns of utilization among the purchaser with 3 or more chronic diseases is significantly no difference after the introduction of cost-sharing in terms of both the number of inpatient days per person and the outpatient visit per person. These results implied that the policy on copayment may result in different patterns of health care utilization according to a proxy as the disease severity of patients.

Before the policy change, the decreasing health care utilization was anticipated among those with supplementary PHI. Although the increasing cost-sharing affected the utilization of outpatient care, these findings do not explain that there was over utilization or that services were appropriately used before policy reform. We could not certainly explain whether purchasers unnecessarily used more health care resources than non-purchaser before the introduction of policy. Also, we could not know change in pattern of outpatient visits among purchasers is due to decreasing unnecessary utilization. However, our findings at least show that the patients with three or more chronic disease, whom are likely to need the addressed health care, have not changed their utilization patterns after the policy implementation. Further, given that the poor people usually were unhealthy, copayment policy led to inhibit the access of health care among these vulnerable groups.

This study has several limitations. First, if individuals who purchased PHI before 2009 renewed their contract with a PHI provider, the prior policy was applied to the contract renewal, as the 2009 change was not retroactive. However, this study could not distinguish between new members and renewal members. Second, this study focused on the effect of health care utilization by both inpatients and outpatients. However, we could not account for the effect of changes in deductibles applied to the cost of drugs. An analysis of changes in the utilization of drugs should be performed. Third, the outcome variables were related to health care utilization, comprising the number of visits and length of stay. These indicators were rather broad, making it difficult to explain the consequences of our study, as health care utilization differs from specific diseases (e.g., a life-threatening situation versus a minor health problem). Fourth, the intervention events must be exempt, and other characteristic should be similar between the groups when using the difference in difference approach. However, the purchasers with indemnity PHI were healthier and younger and had a higher household income than non-purchaser. Thus, the estimate of policy effect could be biased and different characteristic might have affected the observed utilization pattern. These limitations will be considered in a future study.

## Conclusion

In conclusion, the impact of cost-sharing on health care utilization has been well established. In our study, there were two sides effect of cost-sharing in indemnity health insurance. Overall utilization of outpatients decreased after the introduction of cost-sharing in indemnity health insurance; however, this effect size was stronger for the poor and patients with three or more chronic diseases have not changed their health care utilization. Although the introduction of cost-sharing in indemnity health insurance was designed to restrict health care expenditure by reducing moral hazard in medical utilization, cost-sharing lead to reduced access to medical service utilization for the poor. Thus, these results implied meaningful lessons for decision-makers and future health insurance policies in Korea and other countries in terms of cost-sharing in medical care. When policy makers intend to implement the cost-sharing, a different copayment scheme is needed according to the socioeconomic status or disease severity. It has been shown that people in vulnerable groups are more susceptible to lowered access when implementing cost-sharing [39]. In line with previous studies, our results also showed similar trends. Hence, when designing the cost-sharing scheme, we suggest that low rate of cost-sharing in indemnity health insurance may be applied to the poor or persons who need health care (i.e., persons who had many disease or severe disease) so that people of low socioeconomic status or those whom essentially require health care utilization are not hindered from medical access.
